# Oxidative stress impairs energy metabolism in primary cells and synovial tissue of patients with rheumatoid arthritis

**DOI:** 10.1186/s13075-018-1592-1

**Published:** 2018-05-29

**Authors:** Emese Balogh, Douglas J. Veale, Trudy McGarry, Carl Orr, Zoltan Szekanecz, Chin-Teck Ng, Ursula Fearon, Monika Biniecka

**Affiliations:** 1 Department of Rheumatology, University of Debrecen Medical and Health Science Centre, 98. Nagyerdei krt, Debrecen, Hungary; 2 Centre for Arthritis and Rheumatic Diseases, Dublin Academic Medical Centre, St. Vincent’s University Hospital, Dublin, Ireland; 3 Molecular Rheumatology, Trinity Biomedical Sciences Institute Trinity College Dublin, Dublin, Ireland; 4 Department of Rheumatology and Immunology, Singapore General Hospital, Singapore, Singapore; 5 Duke-NUS Medical School, Singapore, Singapore

**Keywords:** Bioenergetic metabolism, Oxidative stress, Angiogenesis, Rheumatoid arthritis

## Abstract

**Background:**

In this study, we examined the effect of oxidative stress on cellular energy metabolism and pro-angiogenic/pro-inflammatory mechanisms of primary rheumatoid arthritis synovial fibroblast cells (RASFC) and human umbilical vein endothelial cells (HUVEC).

**Methods:**

Primary RASFC and HUVEC were cultured with the oxidative stress inducer 4-hydroxy-2-nonenal (4-HNE), and extracellular acidification rate, oxygen consumption rate, mitochondrial function and pro-angiogenic/pro-inflammatory mechanisms were assessed using the Seahorse analyser, complex I–V activity assays, random mutation mitochondrial capture assays, enzyme-linked immunosorbent assays and functional assays, including angiogenic tube formation, migration and invasion. Expression of angiogenic growth factors in synovial tissue (ST) was assessed by IHC in patients with rheumatoid arthritis (RA) undergoing arthroscopy before and after administration of tumour necrosis factor inhibitors (TNFi).

**Results:**

In RASFC and HUVEC, 4-HNE-induced oxidative stress reprogrammed energy metabolism by inhibiting mitochondrial basal, maximal and adenosine triphosphate-linked respiration and reserve capacity, coupled with the reduced enzymatic activity of oxidative phosphorylation complexes III and IV. In contrast, 4-HNE elevated basal glycolysis, glycolytic capacity and glycolytic reserve, paralleled by an increase in mitochondrial DNA mutations and reactive oxygen species. 4-HNE activated pro-angiogenic responses of RASFC, which subsequently altered HUVEC invasion and migration, angiogenic tube formation and the release of pro-angiogenic mediators. In vivo markers of angiogenesis (vascular endothelial growth factor, angiopoietin 2 [Ang2], tyrosine kinase receptor [Tie2]) were significantly associated with oxidative damage and oxygen metabolism in the inflamed synovium. Significant reduction in ST vascularity and Ang2/Tie2 expression was demonstrated in patients with RA before and after administration of TNFi.

**Conclusions:**

Oxidative stress promotes metabolism in favour of glycolysis, an effect that may contribute to acceleration of inflammatory mechanisms and subsequent dysfunctional angiogenesis in RA.

**Electronic supplementary material:**

The online version of this article (10.1186/s13075-018-1592-1) contains supplementary material, which is available to authorized users.

## Background

Angiogenesis is one of the earliest events in the development of rheumatoid arthritis (RA). New blood vessels invade the synovial membrane, resulting in a self-perpetuating and persistent infiltration of immune cells into the joint, transforming the synovial tissue (ST) into an aggressive, tumour-like ‘pannus’ [1]. New capillaries also facilitate the delivery of sufficient oxygen and nutrients to support the proliferating synovium. Although angiogenesis is a prominent feature of RA, the neovascular network is dysfunctional and fails to restore tissue oxygen homeostasis, rendering the inflamed ST hypoxic. The increase in metabolic turnover of the expanding synovial pannus outpaces the oxygen supply, resulting in a demand for adenosine triphosphate (ATP) and an altered regulation of cellular metabolic mechanisms [2, 3].

Bioenergetics is fundamentally important for all cells to enable proliferation, differentiation and maturation, with mitochondria being central to biosynthetic and bioenergetic pathways mediated by the tricarboxylic acid (TCA) cycle. Thus, alterations to mitochondrial respiration can play a key role in mediating pathogenic mechanisms in chronic inflammatory diseases [4–6]. One well-known example of mitochondrial dysfunction is the bioenergetic switch in cell metabolism from oxidative phosphorylation (OXPHOS) towards aerobic glycolysis, known as the Warburg effect. Although the efficiency of ATP production per molecule of glucose is much lower through glycolysis, the yield rate is much faster than that of OXPHOS, supporting rapid cellular growth. It has been demonstrated that the Warburg effect is present in highly proliferating and metabolically active immune cells in a manner similar to that observed in tumour cells. In the inflamed joint, an increase in the metabolic state towards glycolysis has been shown in primary rheumatoid arthritis synovial fibroblasts (RASFC), CD4 T cells, T-helper type 17 (T_H_17) cells, macrophages and dendritic cells [7–10]. This is paralleled by elevated lactate levels and diminished glucose in RA synovial fluids as well as by increased activity of key glycolytic enzymes in the RA synovium, indicating that anaerobic glycolysis is favoured in this hypoxic environment [11–13]. More recently, in vitro studies by our group have shown that hypoxia and Toll-like receptor 2 (TLR2)-induced inflammation promoted mitochondrial dysfunction and oxidative stress and reprogrammed the nature of cellular respiration in RA synovial cells [14, 15].

Oxidative damage occurs through the detrimental effect of hypoxia and is recognised as an important source of genomic instability that leads to respiratory alterations. Hypoxia promotes overproduction of reactive oxygen species (ROS) that provoke oxidation of polyunsaturated fatty acids in plasma and mitochondrial membranes. This generates an array of primary lipid peroxidation products, which subsequently decompose and form reactive lipid electrophiles, among which 4-hydroxy-2-nonenal (4-HNE) is the most important signalling molecule [16]. 4-HNE can form covalent adducts with DNA, phospholipids and nucleophilic amino acids, impairing their structure and biological properties. In particular, mitochondria have been reported as a prominent target of 4-HNE activity [17]. Mitochondrial proteins related to mitochondrial energy metabolism, such as adenosine triphosphate synthase subunit β (ATP5B), succinate dehydrogenase flavoprotein subunit and reduced form of nicotinamide adenine dinucleotide (NADH) dehydrogenase iron–sulphur protein 2 in the electron transport chain (ETC), and trifunctional enzyme subunit α in the TCA cycle, are highly susceptible to 4-HNE-induced inactivation [18–20]. A recent study has also demonstrated 4-HNE-induced inhibition of sirtuin 3, a major mitochondrial nicotinamide adenine dinucleotide (NAD^+^)-dependent deacetylase, with subsequent up-regulation of vascular endothelial growth factor (VEGF) expression by breast cancer cells [21], indicating a close connection between oxidative stress, mitochondrial function and angiogenesis.

In previous studies, our group assessed levels of synovial lipid peroxidation in patients with RA and demonstrated a significant inverse correlation between 4-HNE expression and oxygen tension in the inflamed joint, reflecting mitochondrial damage [22]. Subsequently, we have demonstrated that high synovial lipid peroxidation positively correlated with clinical disease activity scores, and we have reported reduced 4-HNE levels in patients with RA who responded to tumour necrosis factor (TNF) blocking therapy corresponding with a significant increase in partial oxygen pressure in synovial tissue, indicating a reduction in synovial oxidative stress as the joint tissue becomes less hypoxic [23]. In addition, it was observed that increased synovial inflammation and angiogenesis was associated with higher oxidative stress [22]. Given the important role of mitochondrial metabolism in the regulation of inflammatory and angiogenic responses, in this study we investigated the effect of oxidative stress on the mitochondrial bioenergetic profile and the pro-angiogenic/pro-inflammatory mechanisms in RASFC and human umbilical vein endothelial cells (HUVEC). Furthermore, we determined the effects of tumour necrosis factor α inhibitors (TNFi) on the expression of angiogenic markers in RA in relation to synovial oxidative stress in vivo.

## Methods

### Patient recruitment, arthroscopy and sample collection

Fifteen patients with active RA were recruited from the Rheumatology Department of St. Vincent’s University Hospital, Dublin, Ireland. All patients gave fully informed written consent approved by the institutional ethics committee, and the research was performed in accordance with the Declaration of Helsinki. Clinical disease activity was assessed with the 28-joint Disease Activity Score (DAS28) using the C-reactive protein level. Under local anaesthesia, all patients with RA underwent arthroscopy of the inflamed knee joint prior to biologic treatment (T0) and a second arthroscopy 3 months after commencement of TNFi (T3). ST biopsies were used for isolation of primary synovial fibroblasts and histological analyses.

### RASFC culture

RASFC biopsies obtained at arthroscopy were digested with 1 mg/ml collagenase type I (Worthington Biochemical, Lakewood, NJ, USA) in Gibco RPMI 1640 medium (Thermo Fisher Scientific, Paisley, UK) for 4 hours at 37 °C in humidified air with 5% CO_2_. Dissociated cells were plated in RPMI 1640 medium supplemented with 10% Gibco FCS (Thermo Fisher Scientific), 20 mM 4-(2-hydroxyethyl)-1-piperazineethanesulfonic acid (Thermo Fisher Scientific), penicillin (100 U/ml), streptomycin (100 U/ml) and amphotericin B (Fungizone 0.25 μg/ml; (Invitrogen, Plymouth, MN, USA). Cells were grown to confluence and used between passages 4 and 7. RASFC were seeded onto 96-well plates and into T25 flasks and cultured in the presence of 4-HNE (2.5 μM; Cayman Chemical, Ann Arbor, MI, USA), a highly reactive end product of lipid peroxidation or vehicle basal medium (0.1% ethanol). The concentration of 4-HNE used in the experiments was based on a cell viability assay and previously published studies [24]. Following stimulation, the effect of amplified oxidative stress on mitochondrial function, cellular metabolism and angiogenic responses was assessed as described below.

### HUVEC culture

HUVEC (Lonza, Walkerville, MD, USA) were incubated in MCDB (Thermo Fisher Scientific) supplemented with l-glutamine (Thermo Fisher Scientific), 0.5 ml epidermal growth factor (Thermo Fisher Scientific), 50 ml FCS (Thermo Fisher Scientific), 0.5 ml of hydrocortisone, penicillin (100 U/ml; Bioscience), streptomycin (100 U/ml; Bioscience) and Fungizone (0.25 μg/ml; Bioscience). Cells were cultured at 37 °C in humidified air with 5% CO_2_ and harvested with trypsin-ethylenediaminetetraacetic acid (Lonza). Cells were used between passages 20 and 30.

### Oxygen consumption rate and extracellular acidification rate measured using Seahorse technology

Oxygen consumption rate (OCR) and extracellular acidification rate (ECAR), reflecting OXPHOS and glycolysis, respectively, were measured before and after treatment with oligomycin (2 μg/ml), trifluorocarbonylcyanide phenylhydrazone (FCCP; 5 μM), antimycin A (2 μM) and 2-deoxyglucose (2-DG; 25 mM) using the Seahorse XF24 analyser (Agilent Technologies, Santa Clara, CA, USA). RASFC and HUVEC were seeded at 30,000 cells per well in a Seahorse XF96 cell culture microplate (Agilent Technologies) and allowed to adhere for 24 hours. Cells were rinsed with assay medium (unbuffered DMEM supplemented with 10 mM glucose, 1 mM sodium pyruvate and 2 mM l-glutamine, pH 7.4) before incubation with assay medium for 30 minutes at 37 °C in a non-CO_2_ incubator. Following incubation, cells were stimulated with 4-HNE (2.5 μM) and vehicle basal medium for 2 hours. Four baseline OCR and ECAR measurements were obtained over 28 minutes before injection of specific metabolic inhibitors. Moreover, to challenge the metabolic capacity of the RASFC and HUVEC, three OCR and ECAR measurements were obtained over 15 minutes following injection with oligomycin, FCCP, antimycin A and 2-DG.

### In vitro mitochondrial dysfunction and mitochondrial DNA mutagenesis

ROS production was assessed using the DCFDA Cellular Reactive Oxygen Species Detection Assay Kit (Abcam, Cambridge, UK). RASFC were seeded into clear-bottomed, dark-sided 96-well plates at a density of 2.5 × 10^4^ cells/well and allowed to attach overnight. Cells were washed in 1× buffer and stained with 25 μM 2′,7′-dichlorofluorescin diacetate in 1× buffer for 45 minutes at 37 °C and 5% CO_2_. After staining, cells were washed, treated with 4-HNE and incubated at 37 °C in 5% CO_2_. ROS fluorescence signal was measured using the SpectraMax Gemini system (Molecular Devices, Sunnyvale, CA, USA) with excitation and emission wavelengths of 485 nm and 538 nm, respectively. Mean fluorescence values from four wells for each condition were obtained. To characterise the frequencies of random mutations in RASFC exposed to 4-HNE for 24 hours, we used a mitochondrial random mutation capture assay.

Mitochondrial DNA (mtDNA) was extracted using a previously reported protocol [25]. Following extraction, 10 μg of mtDNA was digested with 100 U of *Taq*^α^I restriction enzyme (New England Biolabs, Ipswich, MA, USA), 1× bovine serum albumin, and a *Taq*^α^I-specific digestion buffer (10 mM Tris HCl, 10 mM MgCl_2,_ 100 mM NaCl, pH 8.4) for 10 hours, with 100 U of *Taq*^α^I added to the reaction mixture every hour. PCR amplification was performed in 25-μl reaction mixtures containing 12.5 μl of 2× SYBR Green Brilliant Master Mix (Stratagene, La Jolla, CA, USA), 0.1 μl of uracil DNA glycosylase (New England Biolabs), 0.7 μl of forward and reverse primers (10 pM/μl; Integrated DNA Technologies, Skokie, IL, USA), and 6.7 μl of H_2_O. The samples were amplified using a Roche LightCycler 480 Instrument (Roche Diagnostics, Indianapolis, IN, USA), according to the following protocol; 37 °C for 10 minutes, 95 °C for 10 minutes, followed by 45 cycles of 95 °C for 15 seconds and 60 °C for 1 minute. Samples were kept at 72 °C for 7 minutes and following melting-curve analysis were immediately stored at − 80 °C. The primer sequences used were as follows: for mtDNA copy number, 5′-ACAGTTTATGTAGCTTACCTCC-3′ (forward) and 5′-TTGCTGCGTGCTTGATGCTTGT-3′ (reverse); for random mutations, 5′-CCTCAACAGTTAAATCAACAAAACTGC-3′ (forward) and 5′-GCGCTTACTTTGTAGCCTTCA-3′ (reverse).

### Examination of mitochondrial complexes I–V activity

Mitochondrial complexes I–V OXPHOS activity assay kits (Abcam) were used to screen the direct effect of 4-HNE on all complexes of the mitochondrial respiratory chain. These assays are performed using whole bovine heart mitochondria, a rich source of OXPHOS complexes. The activity of mitochondrial complexes I–V was measured as per the manufacturer’s instructions. Briefly, OXPHOS complex I (NADH ubiquinone oxidoreductase) catalyses electron transfer from NADH to the electron carrier, ubiquinone, concomitantly pumping protons across the inner mitochondrial membrane. The progression of this reaction was monitored following the oxidation as a decrease in absorbance at optical density (OD) 340 nm. OXPHOS complex II (succinate-coenzyme Q reductase) catalyses electron transfer from succinate to the electron carrier, ubiquinone. The product, ubiquinol, is used by complex III in the respiratory chain, and fumarate is necessary to maintain the TCA cycle. The production of ubiquinol in the presence of 4-HNE was monitored at OD 600 nm. To examine OXPHOS complex III activity, succinate (electron donor of complex II) and oxidised cytochrome c (electron acceptor of complex III) were added to the mitochondria to start the electron transfer reaction that takes place during OXPHOS.

The rate of coupled complex II + III reaction was measured by monitoring the conversion of oxidised cytochrome c into reduced form, observed as an increase in absorbance at OD 550 nm. OXPHOS complex IV (cytochrome c oxidase) transfers electrons from reduced cytochrome c to molecular oxygen and concomitantly pumps protons across the inner mitochondrial membrane. The progression of this reaction was monitored following the oxidation as a decrease in absorbance at OD 550 nm. OXPHOS complex V makes about 95% of a cell’s ATP using energy generated by the proton-motive force and can also function in the reverse direction in the absence of a proton-motive force, hydrolysing ATP to generate adenosine diphosphate (ADP) and inorganic phosphate. The production of ADP by ATP synthase can be coupled to the oxidation of NADH to NAD^+^, and the progress of the coupled reaction in the presence of 4-HNE was monitored as a decrease in absorbance at OD 340 nm. Results were calculated using SoftMax Pro 5.3 microplate analysis software (Molecular Devices). The activity of complexes I, II, IV and V is proportional to the decrease in absorbance, and the linear rate of reduction in absorbance over time was calculated. The activity of complex III is proportional to the increase in absorbance, and the linear rate of increase in absorbance over time was calculated. For each complex, results are graphically demonstrated as the percentage of enzymatic activity in the presence of 4-HNE relative to the percentage of basal activity.

### Quantification of pro-angiogenic mediators in RASFC

To assess the effects of oxidative stress on secretion of VEGF, angiopoietin 2 (Ang2), platelet-derived growth factor subunit B (PDGF-B), basic fibroblast growth factor (bFGF), interleukin (IL)-8, regulated on activation, normal T cell expressed and secreted (RANTES) and intercellular adhesion molecule (ICAM), RASFC were seeded into 96-well plates. Confluent RASFC were serum-starved for 24 hours and then cultured with 4-HNE for 24 hours. Supernatants were harvested, and protein secretion levels were quantified using MSD assays (Meso Scale Discovery, Rockville, MD, USA) or specific enzyme-linked immunosorbent assays (ELISAs) (R&D Systems, Minneapolis, MN, USA).

### Induction of pro-angiogenic mechanisms of HUVEC in response to oxidative stress-activated RASFC

To examine if oxidatively activated RASFC could further affect pro-angiogenic mechanisms of HUVEC, RA fibroblast cells were stimulated with 4-HNE for 24 hours, and conditioned media (CM) were harvested. As a basal medium, we used fibroblast-conditioned media from RASFC cultured in the absence of 4-HNE. Next, the culture of HUVEC was supplemented with 10% fibroblast-conditioned media. To ensure that the effects on HUVEC function were not due to residual 4-HNE in the 10% fibroblast-conditioned media, HUVEC were also cultured with RPMI 1640 medium containing 4-HNE at the same concentration (0.25 μM), which is the same concentration as that in the 10% RASFC CM. Following 24-hour exposure of HUVEC to fibroblast-conditioned media, pro-angiogenic responses of endothelial cells were assessed as described in the subsections that follow.

### HUVEC transwell invasion chambers

BD BioCoat Matrigel invasion chambers (BD Biosciences, Wokingham, UK) were used to examine HUVEC invasion. Cells were seeded at a density of 2.5 × 10^4^ per well in the migration chamber on 8-μm membranes pre-coated with Matrigel. HUVEC media containing 10% fibroblast-conditioned media was placed in the lower well of the chamber, and cells were allowed to migrate for 48 hours. Non-migrating HUVEC were removed from the upper surface by gentle scrubbing. Cells that had invaded were attached to the lower membrane and fixed with 4% paraformaldehyde (PFA) and stained with 0.1% crystal violet. To assess the average number of invading HUVEC, cells were counted in five random high-power fields.

### HUVEC tube formation

Matrigel (50 μl; BD Biosciences, San Jose, CA, USA) was plated in 96-well culture plates after thawing on ice and allowed to polymerise for 30 minutes at 37 °C in humidified air with 5% CO_2_. HUVEC were removed from culture, trypsinised and resuspended at a concentration of 4 × 10^4^ cells/ml in endothelial cell growth medium. Five hundred microliters of cell suspension was added to each chamber in the presence of 10% fibroblast-conditioned media and cultured for 8 hours. The tube analysis was determined from five sequential fields (magnification × 10) with a focus on the surface of the Matrigel by two blinded observers and a connecting branch between two discrete endothelial cells was counted as 1 tube.

### HUVEC wound repair assay

HUVEC were seeded onto 24-well plates and grown to confluence. A single scratch wound was induced through the middle of each well with a sterile pipette tip. Cells were subsequently stimulated for 24 hours with 10% fibroblast-conditioned media. HUVEC migration across the wound margins from 8 hours was assessed and photographed using a phase-contrast microscope. Semi-quantitative analysis of cell repopulation of the wound was assessed. Briefly, images of the scratch wound assays were taken at × 10 magnification. The mean closure of the wound was manually calculated from the average of three individual measurements from each wound. This process was repeated for all technical replicates. Measurement of scratches at time 0 were designated as 100% open. From this, the percentage of closure for all scratches was calculated.

### HUVEC proliferation

A crystal violet cell proliferation assay was used to assess HUVEC proliferation in the presence of RASFC-conditioned media. HUVEC were seeded into 96-well culture plates at a density of 5000 cells/well and left overnight at 37 °C and 5% CO_2_. Next, cells were stimulated with 10% fibroblast-conditioned media for 24 hours. Following cell culture, cells were washed with PBS, fixed in 4% PFA and stained with 1% crystal violet solution. Plates were washed with tap water and then dried overnight. Cells were resuspended in 1% Triton X-100 solution (Sigma-Aldrich, St. Louis, MO, USA), and cell number was measured with a microplate reader at a wavelength of 550 nm.

### Quantification of pro-angiogenic mediators in HUVEC

HUVEC were seeded into 96-well plates and left overnight at 37 °C and 5% CO_2_. The following day, cells were stimulated with 10% fibroblast-conditioned media for 24 hours. Next, supernatants were harvested, and protein secretion levels of Ang2 and PDGF-B were quantified by using a specific ELISA (R&D Systems).

### Immunofluorescence staining of RASFC and synovial tissue

Single-immunofluorescence staining was performed on RASFC following 24-hour cell stimulations with 4-HNE. To visualise immunoexpression of VEGF, cells were fixed in 4% PFA and stained with primary rabbit antibody against VEGF (Abcam). To demonstrate ST co-expression of markers of angiogenesis, oxidative stress and bioenergetics, dual-immunofluorescence staining was performed on cryostat synovial sections. ST sections were fixed with acetone for 10 minutes and co-incubated with primary mouse antibody against human 4-HNE (GENTAUR, Kampenhout, Belgium) and with primary rabbit antibodies against VEGF, Ang2, Tie2, ATP5B and glucose transporter 1 (GLUT1) (all from Abcam), glyceraldehyde 3-phosphate dehydrogenase (GAPDH) (Trevigen, Gaithersburg, MD, USA) and pyruvate kinase isozyme 2 (PKM2) (Abgent, San Diego, CA, USA). Following overnight incubation in a humidified chamber, RASFC and ST samples were incubated with Invitrogen Alexa Fluor 488-conjugated goat Invitrogen Superclonal™ anti-mouse secondary antibody (Thermo Fisher Scientific) and Cy™3–conjugated goat anti-rabbit secondary antibody (Jackson ImmunoResearch, West Grove, PA, USA) for 60 minutes and counterstained with 4′,6-diamidino-2-phenylindole (DAPI) nuclear stain (Sigma-Aldrich) for 10 minutes. Samples were mounted with Molecular Probes antifade mounting medium (Thermo Fisher Scientific) and assessed by immunofluorescence microscopy (Olympus BX51; Olympus, Hamburg, Germany).

### IHC and scoring of synovial tissue

IHC was performed using 7-μm cryostat ST sections and the DAKO ChemMate EnVision kit (Dako/Agilent Technologies, Glostrup, Denmark). Sections were defrosted at room temperature for 20 minutes, fixed in acetone for 10 minutes and washed in PBS for 5 minutes. Non-specific binding was blocked using 1% casein in PBS for 20 minutes. The sections were incubated with rabbit monoclonal primary antibodies against human VEGF, Ang2, Tie2, ATP5B (all from Abcam), GAPDH (Trevigen) and mouse monoclonal antibodies against human 4-HNE (GENTAUR). Immunoglobulin G control antibodies were used as negative controls. Following 1-hour incubation with primary antibody, endogenous peroxidase activity was blocked using 0.3% hydrogen peroxide for 5 minutes. Slides were incubated for 30 minutes with secondary antibody/horseradish peroxidase (Dako/Agilent Technologies). 3,3'-Diaminobenzidine (1:50) was used to visualise staining, and Mayer’s haematoxylin (BDH Laboratories, Poole, UK) was incubated for 30 seconds as a counterstain prior to mounting in DPX mounting media. Slides were scored separately for lining layer (LL), sublining layer (SL) and vascular region (BV) using a well-established and validated semi-quantitative scoring method [26], where the percentage of cells that were positive for a specific marker was compared with the percentage of cells that were negative. Percentage positivity was graded using a 0–4 scale, where 0 = no stained cells, 1 = 1–25%, 2 = 25–50%, 3 = 50–75 and 4 = 75–100% stained cells. Images were captured using an Olympus DP50 light microscope and AnalySIS software (Olympus Soft Imaging Solutions, Lakewood, CO, USA).

### Statistical analysis

IBM SPSS Statistics version 20 for Windows software (IBM, Armonk, NY, USA) was used for statistical analysis. Wilcoxon’s signed-rank test, Spearman’s rank-correlation coefficient and the Mann-Whitney *U* test were used for analysis of non-parametric data. Parametric data were analysed using one-way analysis of variance. All *p* values were two-sided, and *p* values less than 0.05 were considered statistically significant.

## Results

### Oxidative stress alters cellular bioenergetics in RASFC and HUVEC in vitro

**Fig. 1:**
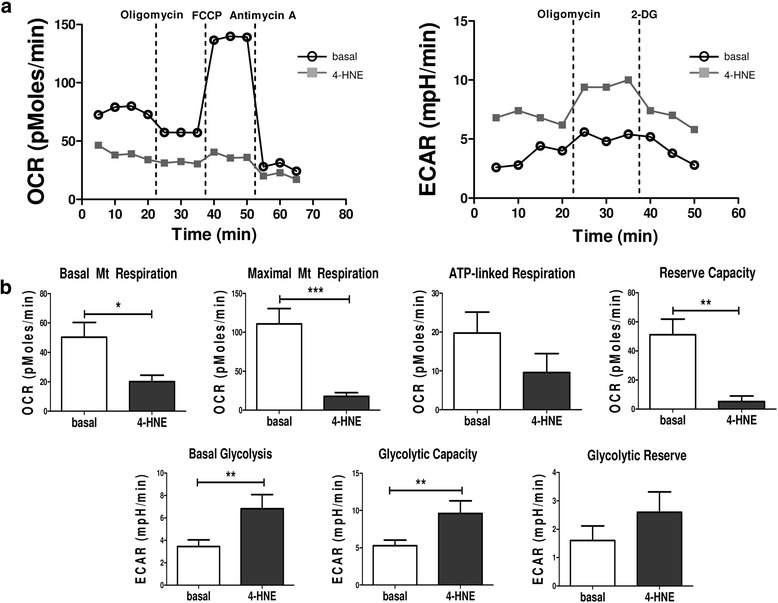
Bioenergetic metabolism in primary rheumatoid arthritis synovial fibroblast cells (RASFC) subjected to 4-hydroxy-2-nonenal (4-HNE)-induced oxidative stress. **a** Representative oxygen consumption rate (OCR) and extracellular acidification rate (ECAR) Seahorse analyser profiles before and after injections of oligomycin, trifluorocarbonylcyanide phenylhydrazone (FCCP), antimycin A and 2-deoxyglucose (2-DG) in RASFC in the presence and absence of 4-HNE. **b** Bar graphs demonstrate quantification of basal mitochondrial (Mt) respiration, maximal Mt respiration, adenosine triphosphate (ATP) synthesis, reserve capacity, basal glycolysis, glycolytic capacity and glycolytic reserve in RASFC (*n* = 5) subjected to oxidative stress. Data are presented as mean ± SEM. *p < 0.05, **p < 0.01, and ***p < 0.001, significant differences from basal level

**Fig. 2:**
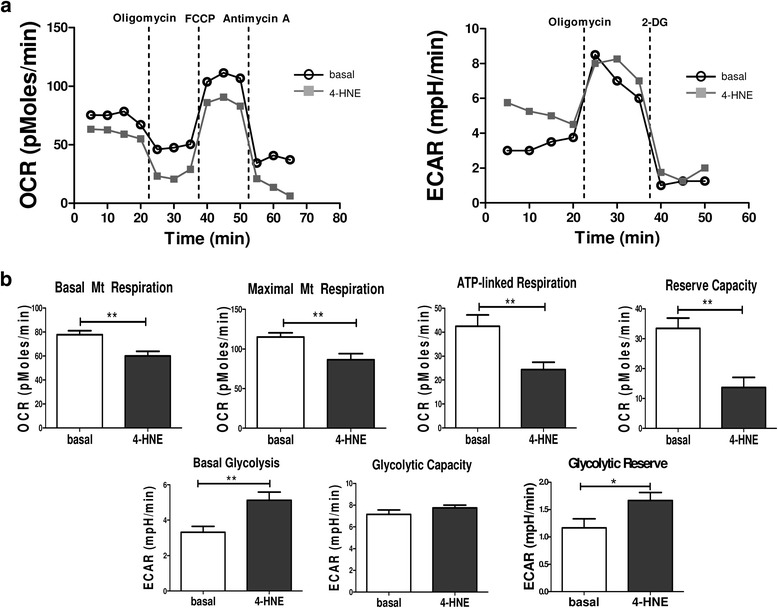
Bioenergetic metabolism in human umbilical vein endothelial cells (HUVEC) subjected to 4-hydroxy-2-nonenal (4-HNE)-induced oxidative stress. **a** Representative oxygen consumption rate (OCR) and extracellular acidification rate (ECAR) Seahorse analyser profiles before and after injections of oligomycin, trifluorocarbonylcyanide phenylhydrazone (FCCP), antimycin A and 2-deoxyglucose (2-DG) in HUVEC in the presence and absence of 4-HNE. **b** Bar graphs demonstrate quantification of basal mitochondrial (Mt) respiration, maximal Mt respiration, adenosine triphosphate (ATP) synthesis, reserve capacity, basal glycolysis, glycolytic capacity and glycolytic reserve in HUVEC (*n* = 3) subjected to oxidative stress. Data are presented as mean ± SEM. *p < 0.05 and **p < 0.01, significant differences from basal level

Previous studies by our group demonstrated altered cellular bioenergetics in RASFC in the presence of hypoxia [14], and we have also demonstrated high oxidative stress in the inflamed synovium [22]. Therefore, in this study, we further investigated whether oxidative stress in the inflamed joint is involved in metabolic reprogramming of RASFC and HUVEC. Figure 1a demonstrates representative OCR and ECAR profiles before and after injections of oligomycin, FCCP, antimycin A and 2-DG in basal and 4-HNE-stimulated RASFC. We show, for the first time to our knowledge, that inhibition of OCR following 4-HNE-induced oxidative stress was associated with a shift in RASFC metabolism towards glycolysis. 4-HNE reduced basal mitochondrial respiration (*p* < 0.05), paralleled by a reduction in maximal mitochondrial respiration (*p* < 0.001), ATP synthesis (*p* = 0.1) and reserve capacity (*p* < 0.01) (Fig. 1b). This metabolic reprogramming was further accompanied by increased levels of basal glycolysis (*p* < 0.01), glycolytic capacity (*p* < 0.01) and glycolytic reserve (*p* = 0.2) in RASFC subjected to oxidative stress (Fig. 1b). Representative HUVEC OCR and ECAR profiles before and after injections of oligomycin, FCCP, antimycin A and 2-DG are shown on Fig. 2a. Similarly to RASFC, 4-HNE inhibited basal mitochondrial respiration, maximal mitochondrial respiration, ATP synthesis and reserve capacity (all *p* < 0.01) with concomitant elevation of basal glycolysis (*p* < 0.01) and glycolytic reserve (*p* < 0.05) in HUVEC exposed to oxidative stress (Fig. 2b).

### Examination of mitochondrial mutagenesis and activity of enzymes of mitochondrial OXPHOS complexes under 4-HNE-induced oxidative stress

**Fig. 3:**
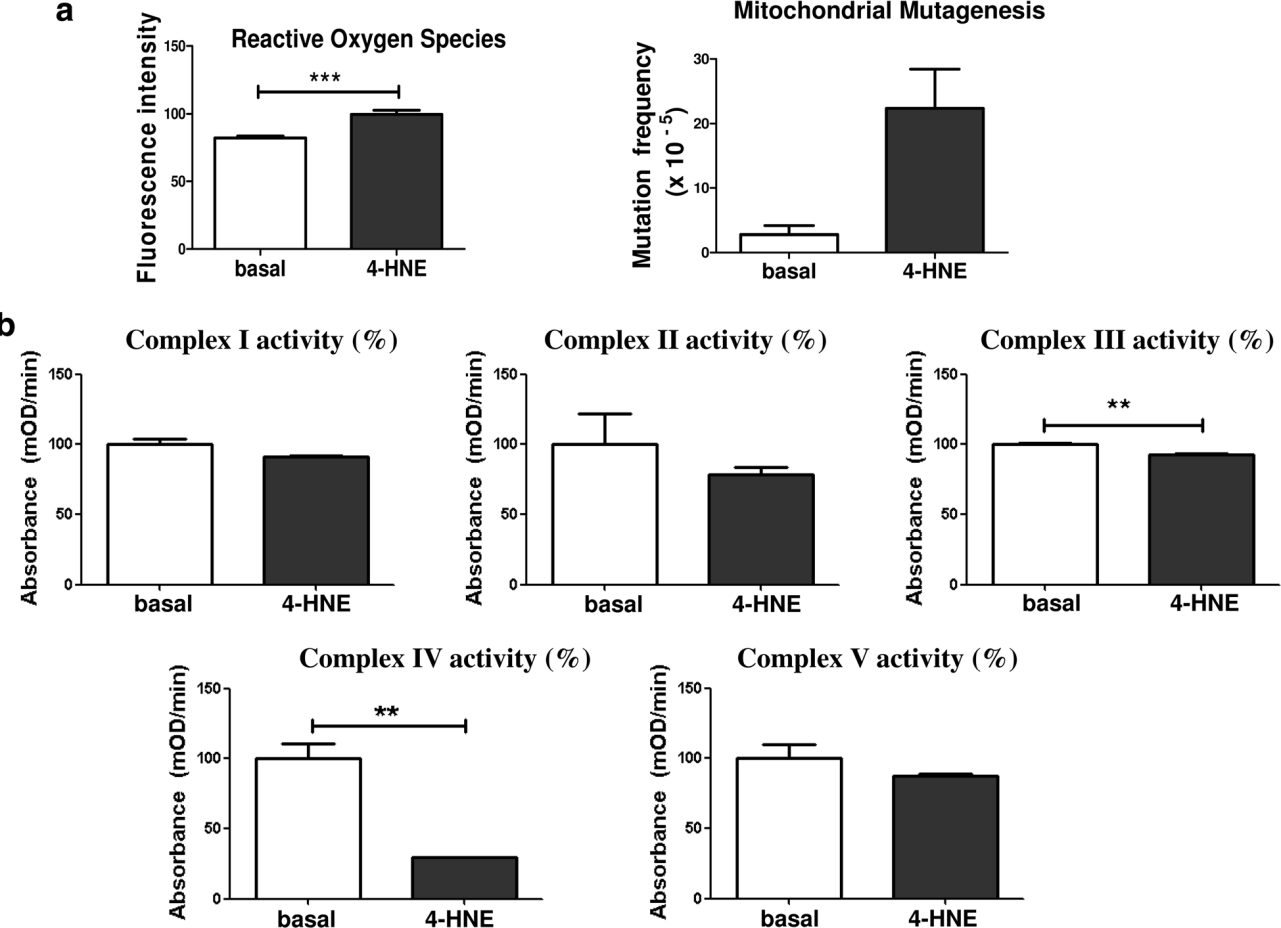
Mitochondrial mutagenesis and activity of enzymes of mitochondrial oxidative phosphorylation (OXPHOS) complexes under 4-hydroxy-2-nonenal (4-HNE)-induced oxidative stress. **a** Bar graphs demonstrate increased production of reactive oxygen species (*n* = 7), paralleled by the greater frequency of mitochondrial DNA mutation (*n* = 5) in primary rheumatoid arthritis synovial fibroblast cells (RASFC) in response to 4-HNE. **b** Activity of mitochondrial OXPHOS complexes I–V in the presence of 4-HNE. 4-HNE reduces the activity of complex I by 9%, complex II by 22%, complex III by 8%, complex IV by 70% and complex V by 12% (all complexes measured in triplicate). For each complex, results are graphically demonstrated as the percentage of enzymatic activity in the presence of 4-HNE relative to the percentage of basal activity. Data is represented as Mean ± SEM, **p<0.01; ***p<0.001 significantly different to basal

We have previously shown that increased mtDNA mutation frequency and mitochondrial dysfunction in the RA joint were strongly associated with synovial inflammation and hypoxia [27, 28]. We have also reported, at a functional level, induction of pro-angiogenic responses of endothelial cells in the presence of oxidative stress [29]. In the present study, we assessed the frequency of mtDNA mutations and mitochondrial dysfunction in RASFC subjected to 4-HNE. We observed increases in ROS production and mtDNA point mutations in RASFC in the presence of 4-HNE compared with basal cells (*p* < 0.001 and *p* = 0.06, respectively) (Fig. 3a). 4-HNE protein adduction may alter protein activity; therefore, we next examined the activity of the individual proteins of mitochondrial OXPHOS complexes I–V. 4-HNE significantly reduced the activity of complex III by 8% and complex IV by 70% compared with basal values (both *p* < 0.01). Lower enzymatic activity following 4-HNE stimulation was also detected for complex I by 9%, complex II by 22% and complex V by 12% (all *p* = 0.2) (Fig. 3b).

### In vitro secretion of pro-angiogenic and pro-inflammatory mediators under oxidative stress conditions

**Fig. 4:**
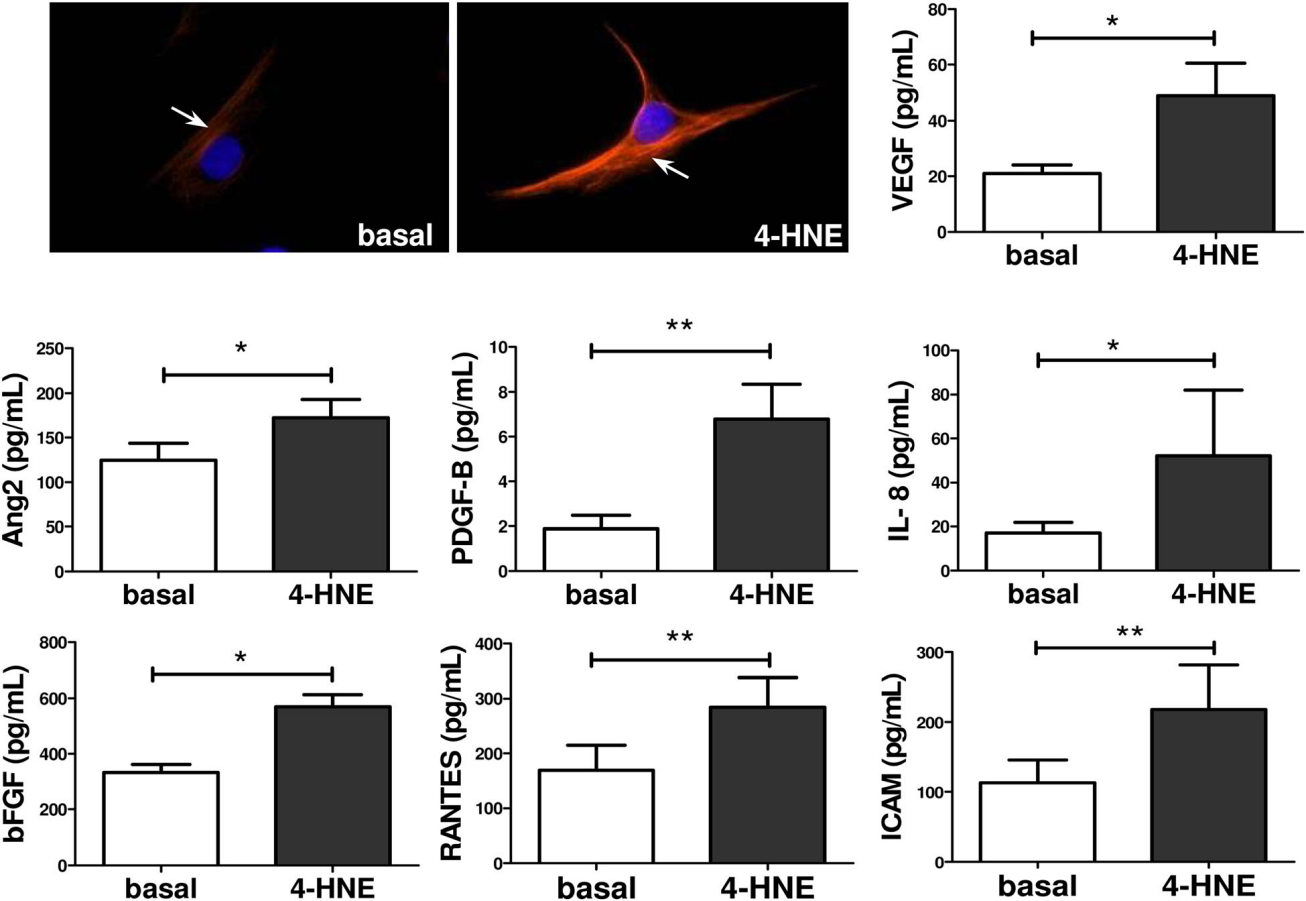
4-Hydroxy-2-nonenal (4-HNE) induces pro-angiogenic and pro-inflammatory mechanisms in primary rheumatoid arthritis synovial fibroblast cells (RASFC). Increased vascular endothelial growth factor (VEGF) immunofluorescence in RASFC subjected to 4-HNE compared to the basal cells and quantification of VEGF, angiopoietin 2 (Ang2), basic fibroblast growth factor (bFGF), interleukin (IL)-8, platelet-derived growth factor subunit B (PDGF-B), regulated on activation, normal T cell expressed and secreted (RANTES), intercellular adhesion molecule (ICAM) in RASFC supernatants (*n* = 7) following cell culture with 4-HNE. Data are presented as mean ± SEM. *p < 0.05 and **p < 0.01, significant differences from basal level. *Red* = VEGF; *blue* = 4′,6-diamidino-2-phenylindole–; magnification of photomicrographs × 40

Because we found a close association of redox state with energy metabolism in RASFC*,* we next examined the effect of oxidative stress on angiogenic and inflammatory mediators from RASFC. Figure 4 demonstrates increased VEGF immunofluorescence staining in RASFC cultured in the presence of 4-HNE compared with the basal cells. In addition, 4-HNE significantly increased secretion of key pro-inflammatory and pro-angiogenic mediators compared with basal RASFC (VEGF, Ang2, bFGF, IL-8 [all *p* < 0.05], PDGF-B, RANTES, ICAM [all *p* < 0.01]). These findings, along with our previously published in vitro study showing TNF-α-induced mitochondrial dysfunction [28], further support the concept of the complex interplay between oxidative damage, oxygen metabolism and angiogenesis in RA. Therefore, we next determined angiogenic in vivo responses following TNFi in 15 patients with RA at baseline (T0) and 3 months after the commencement of biologic treatment (T3). Additional file 1: Figure S1A shows changes of macroscopic vascularity and ST expression of VEGF, Ang2 and Tie2 from T0 to T3. Additional file 1: Figure S1B graphically illustrates decreases in ST VEGF (*p* = 0.1), Ang2 (*p* < 0.005) and Tie2 (*p* < 0.005) after TNFi therapy.

### Oxidative stress-activated RASFC promote pro-angiogenic mechanisms in HUVEC

**Fig. 5:**
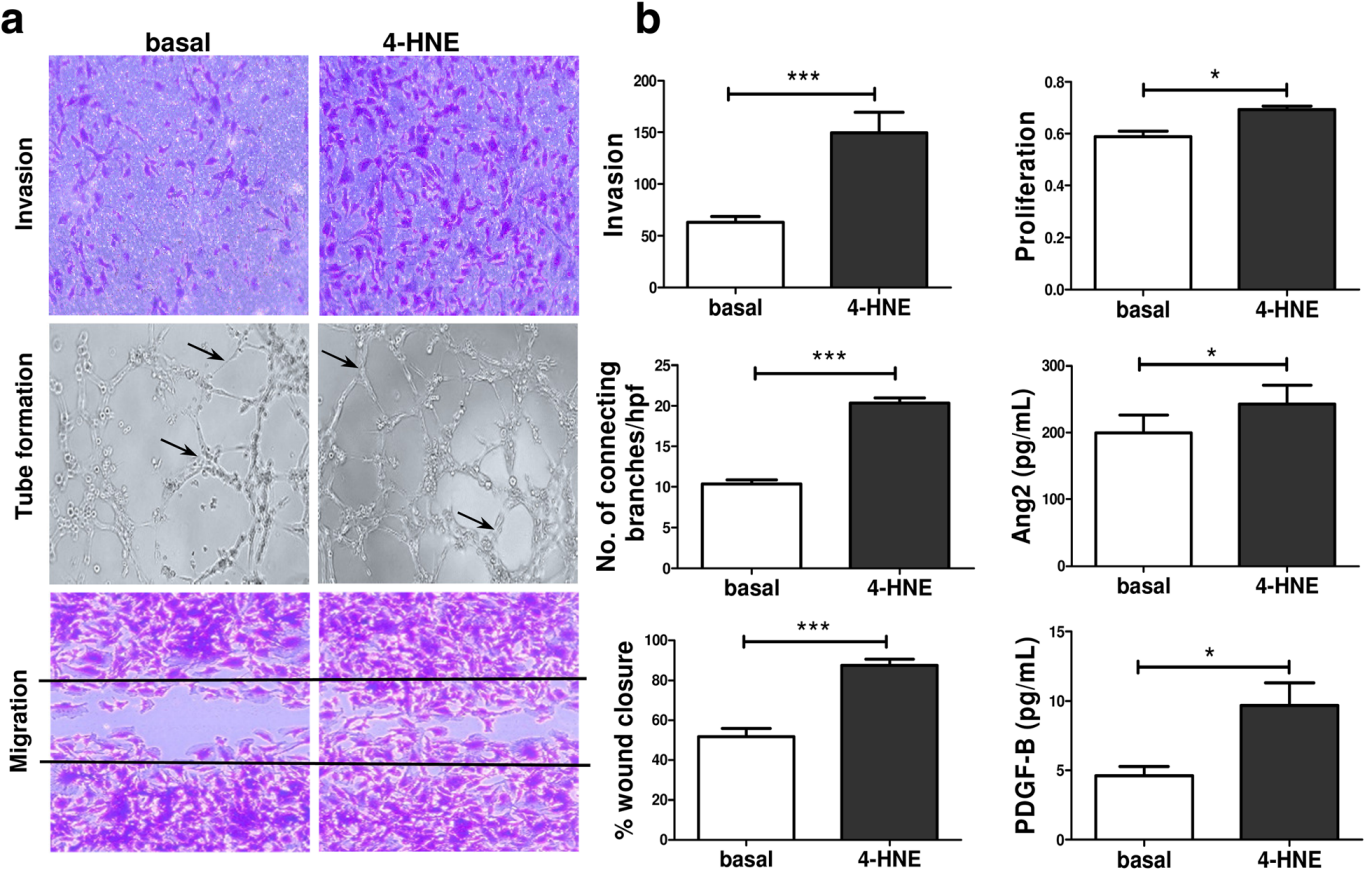
Effects of primary rheumatoid arthritis synovial fibroblast cell (RASFC)-conditioned media on angiogenic responses of human umbilical vein endothelial cells (HUVEC). **a** Representative images demonstrating invasion, the formation of tube-like structures and migration of HUVEC cultured in the presence of basal or 4-hydroxy-2-nonenal (4-HNE)-supplemented conditioned media. Magnification × 10 of photomicrographs demonstrating invasion, tube formation (*arrows* indicate connecting branches) and cell migration. **b** Bar graphs demonstrate an increase in the number of invading, proliferating and migrating HUVEC, a higher number of connecting branches formed between HUVEC, and greater angiopoietin 2 (Ang2) and platelet-derived growth factor subunit B (PDGF-B) release from HUVEC exposed to 4-HNE-supplemented conditioned media (*n* = 6). Data are presented as mean ± SEM. *p < 0.05 and ***p < 0.001, significant differences from basal level. *hpf* High-power field

RASFC are known to be strongly involved in regulating pathological angiogenesis in the inflamed joint [30]. Therefore, we next examined if the observed alterations in cellular bioenergetics and pro-inflammatory processes in RASFC in response to oxidative stress could subsequently influence pro-angiogenic mechanisms in HUVEC. We stimulated RASFC in the presence or absence of 4-HNE and harvested the supernatants, termed *conditioned media*. Fig. 5a demonstrates the effect of basal or 4-HNE RASFC-CM on invasion, the formation of tube-like structures and migration of HUVEC. Figure 5b graphically illustrates markedly induced invasion (*p* < 0.001), proliferation (*p* < 0.05), number of formed tube-like structures (*p* < 0.001), cell migration across the wound (*p* < 0.001) and secretion of Ang2 and PDGF-B (both *p* values < 0.05) in HUVEC in response to basal or 4-HNE RASFC-CM. To confirm that the increase in pro-angiogenic responses of HUVEC was due to oxidatively activated RASFC and not to residual 4-HNE present in the CM, additional experiments were performed, consisting of RPMI 1640 media supplemented with 4-HNE (0.25 μM; 4-HNE RPMI 1640 control), which would be at the same concentration of 4-HNE in the 10% RASFC-CM. A significant increase in invasion (*p* < 0.001), number of formed tube-like structures (*p* < 0.01) and cell migration across the wound (p < 0.001) in HUVEC in response to 4-HNE RASFC-CM compared with 4-HNE RPMI 1640 control media further supports the direct effect of 4-HNE on RASFC-induced angiogenesis in the inflamed joint (Additional file 2: Figure S2).

### Association between ST angiogenesis, oxidative stress and bioenergetics

**Fig. 6:**
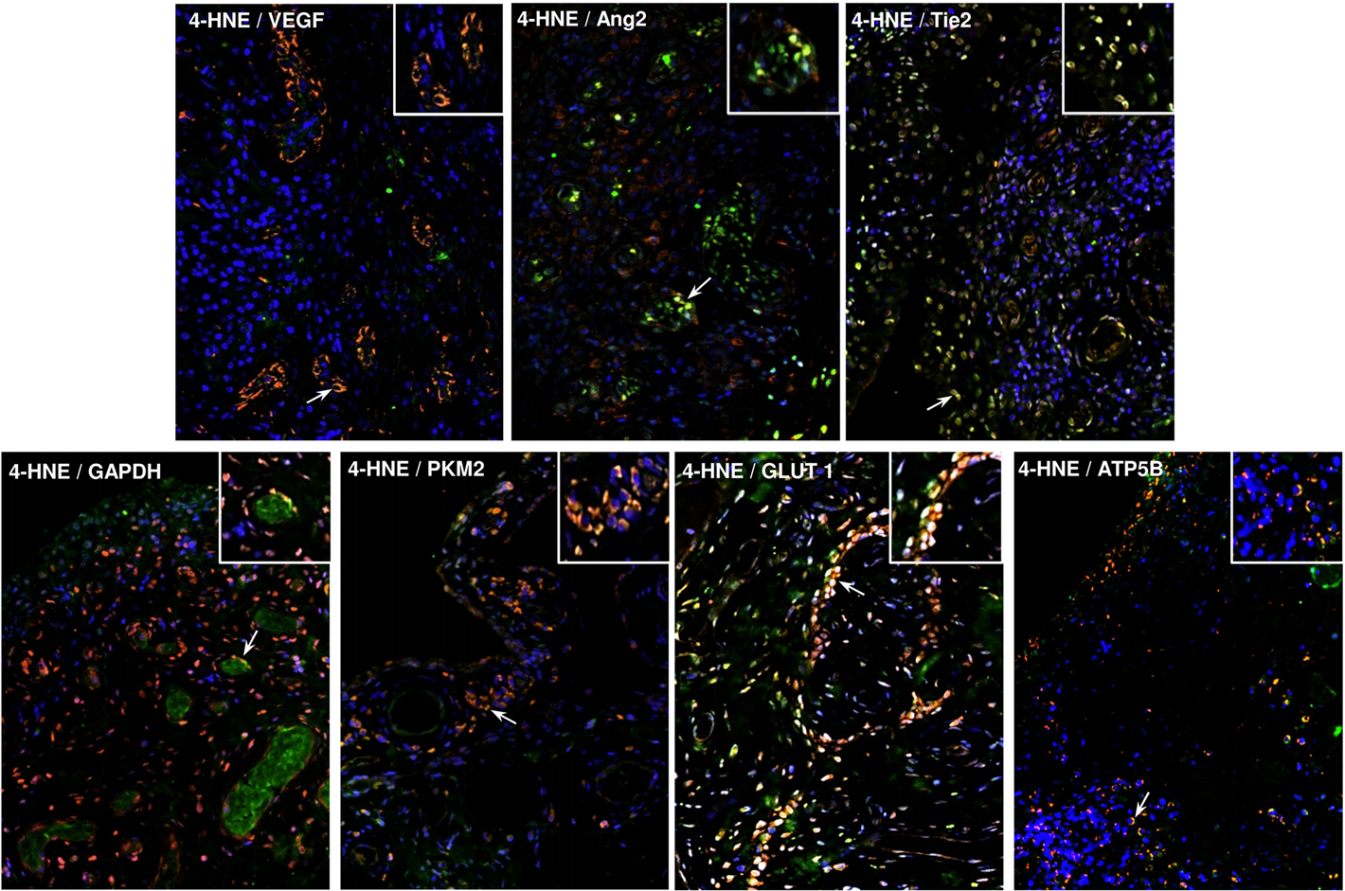
Synovial tissue (ST) angiogenesis, oxidative stress and cellular bioenergetics. To support the concept that oxidative stress, angiogenesis and energy metabolism are interconnected processes that co-exist during the inflammation milieu, double-immunofluorescence staining was performed. ST slides were co-incubated with primary mouse antibody against human 4-hydroxy-2-nonenal (4-HNE) and with primary rabbit antibodies against angiogenic factors (vascular endothelial growth factor [VEGF], angiopoietin 2 [Ang2], tyrosine kinase receptor [Tie2]), glycolytic proteins (glyceraldehyde 3-phosphate dehydrogenase [GAPDH], pyruvate kinase isozyme 2 [PKM2], glucose transporter 1 [GLUT1]) and a mitochondrial marker (adenosine triphosphate synthase subunit β [ATP5B]). Representative merged immunofluorescence images demonstrate examples of co-localisation (*yellow*) of 4-HNE with VEGF, Ang2, Tie2, GAPDH, PKM2, GLUT1 and ATP5B. Cells stained *green* are positive for 4-HNE only; cells stained *red* are positive only for VEGF, Ang2, Tie2, GAPDH, PKM2, GLUT1 and ATP5B. *Arrows* indicate examples of co-localisation. Magnification of photomicrographs × 20, *insets* show high-power magnification of co-localisation. Representative images show single immunofluorescence of 4-HNE, VEGF, Ang2, Tie2, GAPDH, PKM2, GLUT1 and ATP5B along with their controls. Isotype-matched antibodies are shown in Additional file 3: Figure S3 and Additional file 4: Figure S4

Finally, the correlation of angiogenic factors with previously assessed markers of oxidative stress and metabolism in this patient cohort was examined [14]. ST 4-HNE expression was associated with increased expression of VEGF (*r* = 0.63; *p* = 0.015) and Tie2 (*r* = 0.56; *p* = 0.029), GAPDH (*r* = 0.60; *p* = 0.03) and with reduced levels of ATP5B (*p* = − 0.52, *p* = 0.017). Furthermore, representative immunofluorescence images demonstrating co-localisation of 4-HNE with angiogenic factors (VEGF, Ang2, Tie2), as well as with mitochondrial (ATP5B) and glycolytic (GAPDH, PKM2, GLUT1) proteins, is demonstrated in Fig. 6. Additional file 3: Figure S3 and Additional file 4: Figure S4 show single images of VEGF, Ang2, Tie2, GAPDH, PKM2, GLUT1 and ATP5B (all in red), single images of 4-HNE immunofluorescence (in green), as well as single images of DAPI (in blue), along with their controls with isotype-matched antibodies.

## Discussion

In this study, we demonstrate, for the first time to our knowledge, that oxidative stress reprograms cellular bioenergetics of RASFC and HUVEC by downregulating OXPHOS and promoting glycolysis. This change was reflected by a decrease in mitochondrial maximal and ATP-linked respiration and reserve capacity, whereas glycolytic capacity and glycolytic reserve were elevated in the presence of 4-HNE. A bioenergetic switch was coupled with higher ROS production and mtDNA mutations, in addition to the reduced enzymatic activity of mitochondrial complexes III and IV. Oxidative stress also induced secretion of pro-angiogenic and pro-inflammatory mediators by RASFC. CM from 4-HNE-activated RASFC potentiated pro-angiogenic mechanisms in HUVEC, as reflected by elevated cell invasion, proliferation, migration, the formation of tube-like structures and secretion of pro-angiogenic mediators. In vivo co-expression of angiogenic markers, oxidative damage and oxygen metabolism was demonstrated in ST. Finally, a decrease in ST angiogenesis was observed in patients with RA following TNFi therapy.

Hypoxia is a fundamental metabolic change in ST of RA associated with elevated mitochondrial ROS production and lipid peroxidation. Covalent modifications of mtDNA, lipids and proteins by 4-HNE have been reported to compromise mitochondrial integrity and function, including respiratory metabolism, protein transportation, mitochondrial dynamics and quality control through fission, fusion and mitophagy [16]. We have previously shown that increased mtDNA mutation frequency and mitochondrial dysfunction in the RA joint correlated with greater hypoxia, oxidative stress, vascularity and pro-inflammatory cytokines [27, 28]. Our present in vitro findings using RASFC further demonstrate high susceptibility of the mitochondrial genome to oxidative damage. A mitochondrial random mutation capture assay was used to quantify the frequency of random mitochondrial point mutations in RASFC following 4-HNE stimulations. This methodology relies on single-molecule amplification to screen a large number of mtDNA molecules for the presence of unexpanded mutations that may appear following oxidative stress. Elevated mitochondrial mutagenesis detected in RASFC exposed to oxidative stress supports the evidence of the mutagenic nature of 4-HNE. 4-HNE-guanine adducts have been detected in the *p53* tumour suppressor gene in a human lymphoblastoid cell line, causing gene mutation and affecting cell cycle arrest, apoptosis, DNA repair and differentiation [31]. Elevated mtROS levels are considered a primary source of mitochondrial mutagenesis. Our findings show high production of ROS by RASFC exposed to 4-HNE, indicating the ability of 4-HNE to further exacerbate ROS generation, thereby creating the vicious cycle of oxidative stress-induced alteration to the mitochondrial genome.

We investigated if mitochondrial genome instability driven by products of lipid peroxidation is accompanied by defects in respiratory metabolism. The two major energy pathways were measured to find that in the presence of 4-HNE, RASFC and HUVEC switched their bioenergetic profile from OXPHOS to anaerobic glycolysis to respond to an increased energy demand. This was coupled with reduced maximal and ATP-linked respiration and reserve capacity, in contrast to elevated glycolytic capacity and glycolytic reserve. In addition, the enzymatic activities of mitochondrial complexes were decreased by oxidative stress. This compensatory reliance on anaerobic glycolysis may provide a short-term solution; however, prolonged dependence may result in a severe energy deficiency that ultimately creates a bioenergetic crisis, most likely supporting abnormal angiogenesis, cellular invasion and pannus formation.

Our data are consistent with previous studies demonstrating 4-HNE-induced mitochondrial respiration deficiency in cardiac and small airway epithelial cells [32, 33]. Inhibition of mitochondrial respiration following 4-HNE stimulation could be due to reduced functionality from 4-HNE protein-adducts of proteins associated with the ETC and ATP synthase, or it could be due to a diminished ability of RASFC to detoxify 4-HNE because this process requires energy. A study using a proteomic approach identified several 4-HNE-modified mitochondrial proteins in cardiac mitochondria from mice treated with doxorubicin, one of the most widely used chemotherapeutic drugs [34]. Identified proteins were related to mitochondrial energy metabolism, including subunits of the ETC such as NDUFS2 (complex I), SDHA (complex II) and ATP5B (complex V), as well as dihydrolipoamide dehydrogenase, a component of the TCA cycle. Subsequently, 4-HNE adduction reduced enzymatic activity of the mitochondrial proteins, declined OCR and increased ECAR profiles. Other studies identified the 4-HNE modification of proteins involved in metabolism, adhesion, cytoskeletal reorganisation and anti-oxidation in human platelets [35].

In this study, increased secretion of pro-angiogenic and pro-inflammatory mediators by RASFC was observed in the response to 4-HNE. Furthermore, RASFC-CM potentiated pro-angiogenic processes of HUVEC, including endothelial cell invasion, proliferation and migration; the formation of tube-like structures; and secretion of pro-angiogenic mediators. These findings provide evidence for direct and indirect pro-angiogenic effects in response to 4-HNE within the inflamed joint. Our findings are in agreement with other studies showing 4-HNE-induced expression of COX-2, IL-1β, IL-18 and NF-κB and activation of the NLRP3 inflammasome [36–38]. In inflammatory conditions, oxidative stress may mediate angiogenic mechanisms through VEGF-independent pathways involving ROS-induced lipid oxidation. Redox upregulated angiogenic responses of RASFC observed in our study were also reported by others in HUVEC, keratinocytes and epithelial lung and retinal cells [29, 39, 40]. Blocking glycolysis with glycolytic inhibitors reduces pro-inflammatory responses of RASFC and HUVEC as well as the severity of arthritis in K/BxN mice [7, 14]. Furthermore, hypoxic activation of the glycolytic enzyme glucose-6-phosphate isomerase up-regulated VEGF secretion, proliferation and invasion in RASFC and HUVEC [41]. Several glycolytic proteins, including PKM2, GAPDH, fructose bisphosphate aldolase A (aldolase A) and phosphoglycerate kinase 1 have been shown to be adducted by lipid electrophiles [42–44]. Subsequently, this covalent modification is suggested to impair glucose metabolism and result in the accumulation of glycolytic intermediates. This is consistent with studies showing significant increases in lactate levels and ECAR by human platelets cultured with 4-HNE [35] and increased ^18^F-fludeoxyglucose uptake and glycolytic metabolism by oxidised low-density lipoprotein (oxLDL) through upregulation of GLUT1 expression and hexokinase activity [45]. This response was mediated by hypoxia-inducible factor 1α activation and dependent on ROS overproduction. In turn, this metabolic effect of oxLDL was completely abrogated by Src (PP2) and phosphatidylinositol-3 kinase inhibitors, supporting the regulatory role of this pathway in glucose metabolism and immune cell activation.

TNF-α promotes angiogenesis and may regulate capillary formation via VEGF, Ang1 and Ang2 and their receptors. In this study, we investigated whether TNFi therapy alters levels of angiogenic markers 3 months after the commencement of treatment. Following TNFi treatment, reduced ST expression of VEGF, Tie2 receptor and its Ang2 ligand was observed, which further supports the strong link between angiogenesis and TNF-α. These findings are in agreement with those of other studies showing reduced expression of angiogenic markers and endothelial cell activation following TNFi treatment [46, 47]. Additionally, previous studies have demonstrated positive effects of TNFi, including etanercept and infliximab, on oxidative damage in RA, showing significantly reduced serum and urinary levels of oxidative DNA damage and lipid peroxidation with corresponding decreases in DAS28 score following TNFi therapy [48, 49]. Similarly, the serum level of oxidative stress markers was remarkably suppressed in patients with RA treated with tocilizumab IL-6-blocking therapy compared with those treated with anti-TNF antibodies [50].

## Conclusions

In this study, we examined the interplay of synovial cellular bioenergetics, oxidative stress and angiogenesis in RA. We have demonstrated that oxidative stress switched bioenergetic profiles from OXPHOS to anaerobic glycolysis in response to an increased energy demand in the inflamed joint. This creates a bioenergetic crisis that may contribute to dysfunctional angiogenesis to further promote inflammatory mechanisms in RA. In addition, ST upregulation of the angiopoietin/Tie2 system can be altered following TNFi therapy.

## Additional files

Additional file 1: **Figure S1.** Synovial tissue expression of angiogenic markers in patients with RA. **A** Representative images demonstrating macroscopic vascularity and ST VEGF, Ang2 and Tie2 immunostaining at baseline (T0) and 3 months after the commencement of biologic treatment (T3). Magnification of photomicrographs × 20. **B** Baseline and 3 months post-TNFi quantification of ST VEGF, ST Ang2 and ST Tie2 in patients with RA (*n* = 15). Data are presented as mean ± SEM. *p < 0.05; **p < 0.01. (TIF 5466 kb)

Additional file 2: **Figure S2.** Effects of oxidatively activated RASFC on angiogenic responses of HUVEC. To confirm that the increase in pro-angiogenic responses of HUVEC is due to oxidatively activated RASFC and not residual 4-HNE present in the conditioned media, HUVEC were cultured in the presence of RPMI 1640 media supplemented with 4-HNE (0.25 μM; 4-HNE RPMI 1640 control), which was at the same concentration of 4-HNE in the 10% RASFC conditioned media. Representative images and bar graphs demonstrate higher invasion, greater number of formed tube-like structures and greater cell migration across the wound in HUVEC in response to 4-HNE RASFC-conditioned media (4-HNE RASFC-CM; *n* = 6) than in response to 4-HNE RPMI 1640 control. Data are presented as mean ± SEM. **p < 0.01 and ***p < 0.001, representing significant differences from control. Magnification of photomicrographs demonstrating invasion, tube formation (*arrows* show connecting branches) and cell migration × 10. (TIF 8263 kb)

Additional file 3: **Figure S3.** ST angiogenesis and oxidative stress. Representative images of immunofluorescent staining between markers of angiogenesis and 4-HNE in inflamed synovial tissue of patients with RA: VEGF, Ang2 and Tie2 (*red*); 4-HNE (*green*); DAPI (*blue*); and merged images (*yellow*). *Insets* show negative control staining with isotype-matched antibodies. Magnification of photomicrographs × 20. (TIF 9793 kb)

Additional file 4: **Figure S4.** ST cellular bioenergetics and oxidative stress. Representative immunofluorescence images show co-localisation of the oxidative stress marker 4-HNE with glycolytic proteins (GAPDH, PKM2, GLUT1) and a mitochondrial marker (ATP5B) in inflamed ST of patients with RA: GAPDH, PKM2, GLUT1, and ATP5B (*red*); 4-HNE (*green*); DAPI (*blue*); merged images (*yellow*). *Insets* show negative control staining with isotype-matched antibodies. Magnification of photomicrographs × 20. (TIF 9536 kb)

## Abbreviations

2-DG: 2-Deoxyglucose
4-HNE: 4-Hydroxy-2-nonenal
ADP: Adenosine diphosphate
Ang2: Angiopoietin 2
ATP: Adenosine triphosphate
ATP5B: Adenosine triphosphate synthase subunit β
bFGF: Basic fibroblast growth factor
CM: Conditioned media
DAS28: 28-Joint Disease Activity Score
DAPI: 4′,6-Diamidino-2-phenylindole
ECAR: Extracellular acidification rate
ETC: Electron transport chain
FCCP: Trifluorocarbonylcyanide phenylhydrazone
GAPDH: Glyceraldehyde 3-phosphate dehydrogenase
GLUT1: Glucose transporter 1
HUVEC: Human umbilical vein endothelial cells
ICAM: Intercellular adhesion molecule
IL-8: Interleukin 8
Mt: Mitochondrial
mtDNA: Mitochondrial DNA
OCR: Oxygen consumption rate
OD: Optical density
oxLDL: Oxidised low-density lipoprotein
OXPHOS: Oxidative phosphorylation
PDGF-B: Platelet-derived growth factor subunit B
PFA: Paraformaldehyde
PKM2: Pyruvate kinase isozyme 2
RA: Rheumatoid arthritis
RANTES: Regulated on activation, normal T cell expressed and secreted
RASFC: Primary rheumatoid arthritis synovial fibroblast cell
RMC: Random mutation capture assay
ROS: Reactive oxygen species
ST: Synovial tissue
T0: Time point 0 or baseline
T3: Time point 3 months after starting therapy
TCA: Tricarboxylic acid cycle
T_H_17: T-helper type 17 cells
Tie2: Tyrosine kinase receptor
TLR2: Toll-like receptor 2
TNF-α: Tumour necrosis factor α
TNFi: Tumour necrosis factor α inhibitor
VEGF: Vascular endothelial growth factor
