# Supplementary figures and images for: Oxidative stress impairs energy metabolism in primary cells and synovial tissue of patients with rheumatoid arthritis
Source: Arthritis Res Ther. 2018 May 29;20:95. doi: 10.1186/s13075-018-1592-1 (PMC5972404; doi:10.1186/s13075-018-1592-1)

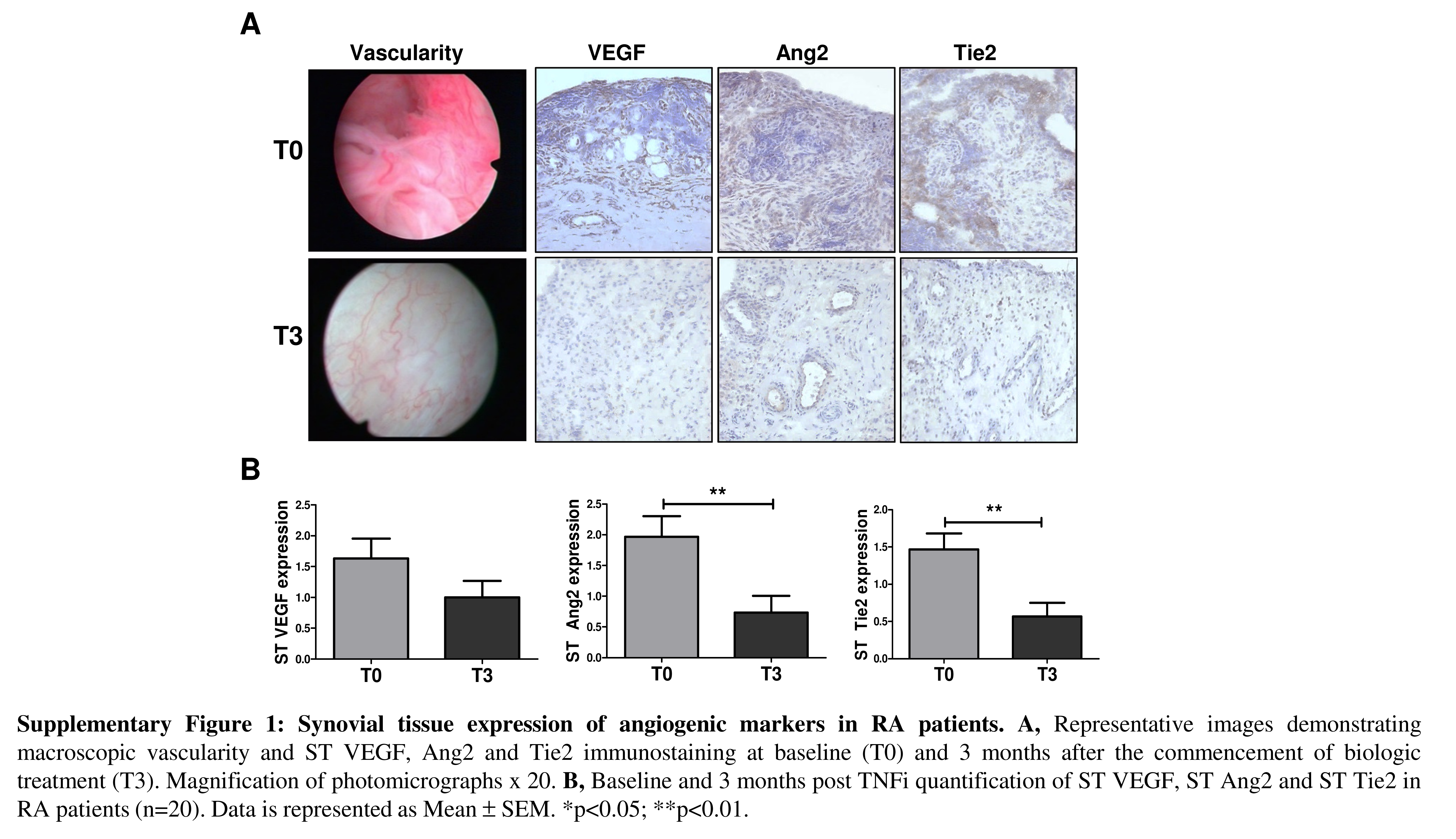

Supplement: Supplementary file 1 — Figure S1. Synovial tissue expression of angiogenic markers in patients with RA. A Representative images demonstrating macroscopic vascularity and ST VEGF, Ang2 and Tie2 immunostaining at baseline (T0) and 3 months after the commencement of biologic treatment (T3). Magnification of photomicrographs × 20. B Baseline and 3 months post-TNFi quantification of ST VEGF, ST Ang2 and ST Tie2 in patients with RA (n = 15). Data are presented as mean ± SEM. *p < 0.05; **p < 0.01. (TIF 5466 kb) [file 13075_2018_1592_MOESM1_ESM.tif]

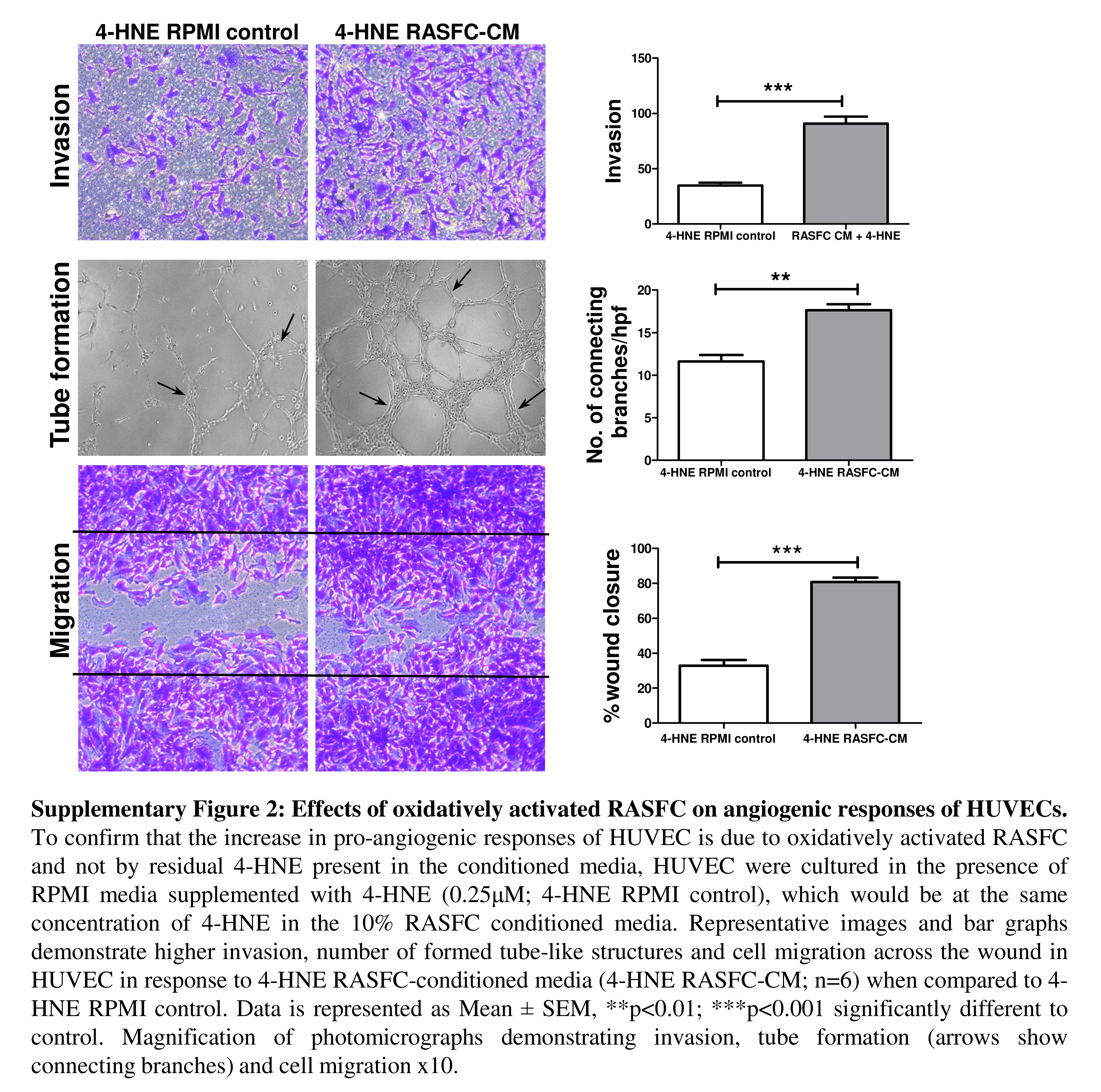

Supplement: Supplementary file 2 — Figure S2. Effects of oxidatively activated RASFC on angiogenic responses of HUVEC. To confirm that the increase in pro-angiogenic responses of HUVEC is due to oxidatively activated RASFC and not residual 4-HNE present in the conditioned media, HUVEC were cultured in the presence of RPMI 1640 media supplemented with 4-HNE (0.25 μM; 4-HNE RPMI 1640 control), which was at the same concentration of 4-HNE in the 10% RASFC conditioned media. Representative images and bar graphs demonstrate higher invasion, greater number of formed tube-like structures and greater cell migration across the wound in HUVEC in response to 4-HNE RASFC-conditioned media (4-HNE RASFC-CM; n = 6) than in response to 4-HNE RPMI 1640 control. Data are presented as mean ± SEM. **p < 0.01 and ***p < 0.001, representing significant differences from control. Magnification of photomicrographs demonstrating invasion, tube formation (arrows show connecting branches) and cell migration × 10. (TIF 8263 kb) [file 13075_2018_1592_MOESM2_ESM.tif]

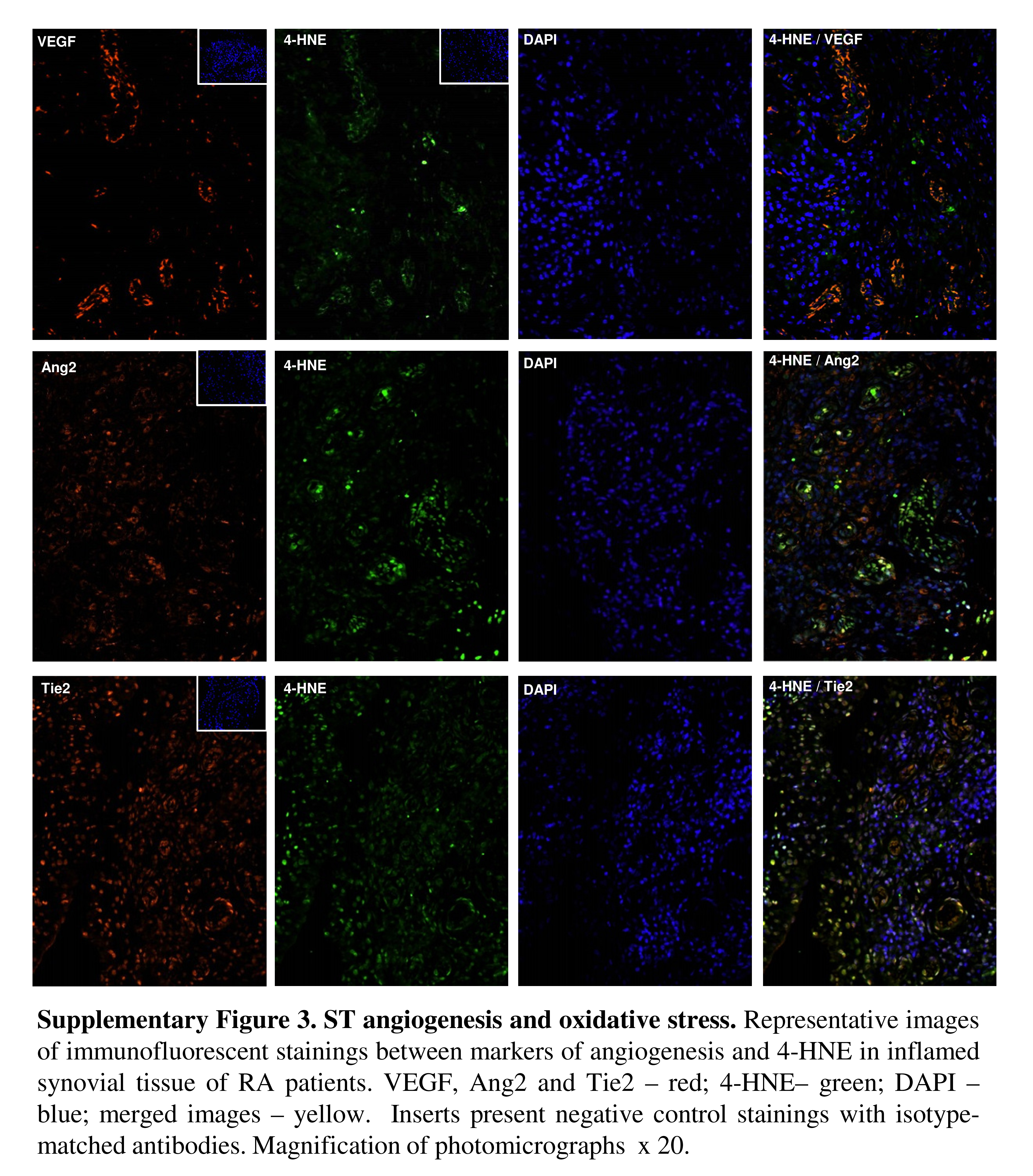

Supplement: Supplementary file 3 — Figure S3. ST angiogenesis and oxidative stress. Representative images of immunofluorescent staining between markers of angiogenesis and 4-HNE in inflamed synovial tissue of patients with RA: VEGF, Ang2 and Tie2 (red); 4-HNE (green); DAPI (blue); and merged images (yellow). Insets show negative control staining with isotype-matched antibodies. Magnification of photomicrographs × 20. (TIF 9793 kb) [file 13075_2018_1592_MOESM3_ESM.tif]

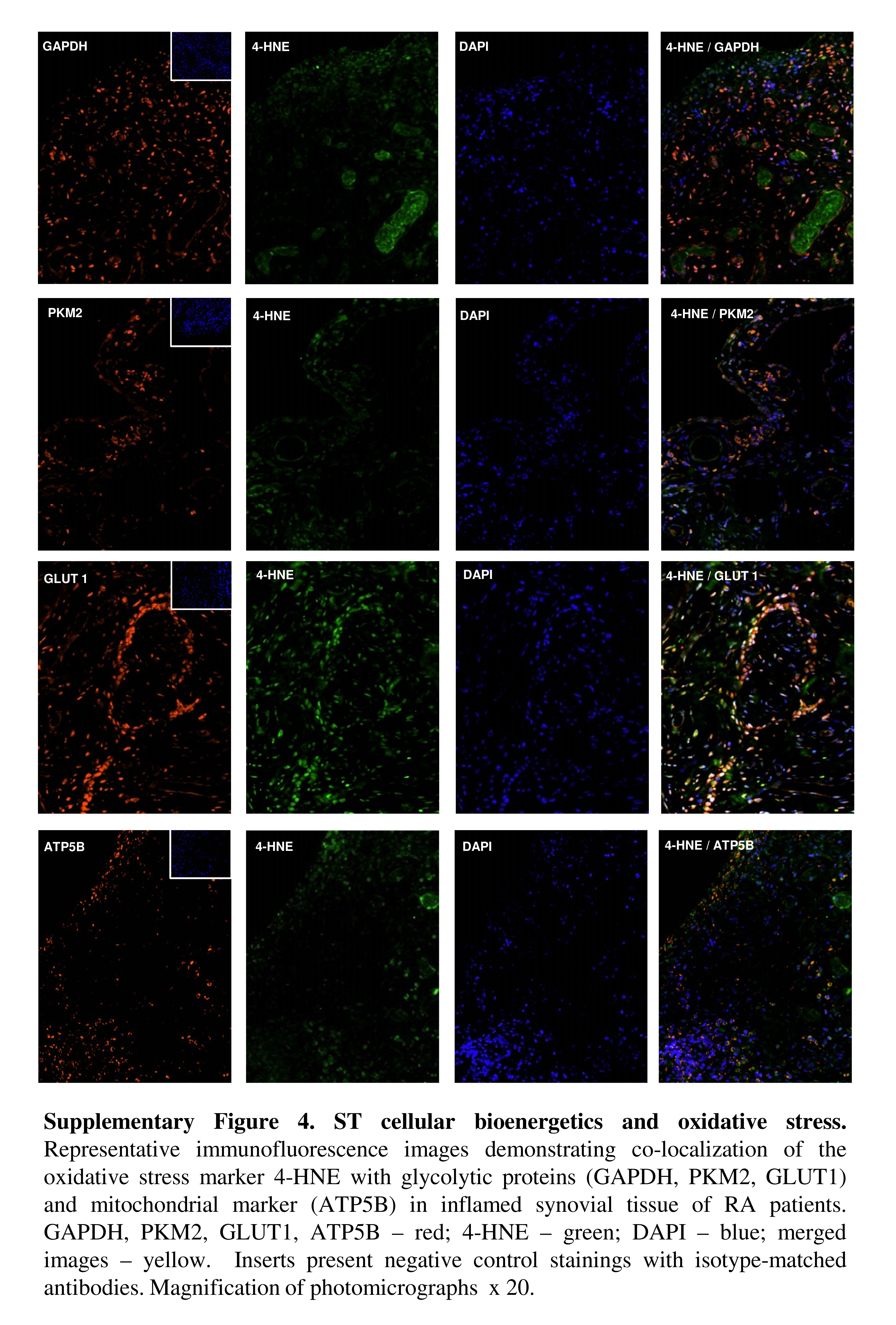

Supplement: Supplementary file 4 — Figure S4. ST cellular bioenergetics and oxidative stress. Representative immunofluorescence images show co-localisation of the oxidative stress marker 4-HNE with glycolytic proteins (GAPDH, PKM2, GLUT1) and a mitochondrial marker (ATP5B) in inflamed ST of patients with RA: GAPDH, PKM2, GLUT1, and ATP5B (red); 4-HNE (green); DAPI (blue); merged images (yellow). Insets show negative control staining with isotype-matched antibodies. Magnification of photomicrographs × 20. (TIF 9536 kb) [file 13075_2018_1592_MOESM4_ESM.tif]
